# Association between *Helicobacter pylori* Infection and Colorectal Adenomatous Polyps

**DOI:** 10.1155/2019/7480620

**Published:** 2019-12-18

**Authors:** Wen Yang, Xueqing Yang

**Affiliations:** ^1^No. 6 Medical Center of the PLA General Hospital, No. 6 Fuchenglu Road, Beijing 100048, China; ^2^Department of Gastroenterology and Hepatolog, Sunshine Union Hospital, Weifang, Shandong Province 261061, China; ^3^Economic Department, University of Miami, 5250 University Dr. Coral Gables, FL 33146, USA

## Abstract

**Background:**

*Helicobacter pylori* infection is a common chronic infection worldwide. At the same time, the incidence of colorectal adenomatous polyps is also at high levels. In order to assess the relationship between *Helicobacter pylori* infection and the occurrence of colorectal adenomatous polyps, we observed 166 patients who had undergone an electronic colonoscopy and ^13^C urea breath test in the outpatient clinic.

**Method:**

A total of 166 (87 males and 79 females, aged 53.85 ± 9.18 years) patients who had colonoscopy examination and ^13^C urea breath test were divided into a *Helicobacter pylori*-positive group (*n* = 68) and *Helicobacter pylori*-negative group (*n* = 98) by the ^13^C urea breath test. At the same time, total cholesterol, triglycerides, and fasting blood sugar were measured and the occurrence of hypertension was counted.

**Results:**

Patients with *Helicobacter pylori* infection had higher incidence of colorectal adenomatous polyps and multiple colorectal adenomatous polyps, higher levels of total cholesterol and fasting glucose, and more males (*P* < 0.05, *P* < 0.01). It was found that *Helicobacter pylori* infection (*P* < 0.05, OR 2.383) was significantly associated with the risk of colorectal adenomatous polyps by binary logistic regression analysis.

**Conclusions:**

Patients with *Helicobacter pylori* infection had higher incidence of colorectal adenomatous polyps.

## 1. Introduction

*Helicobacter pylori* is a common gram-negative bacterium that dwells on the gastric mucosa and can secrete urea enzymes, vacuolus toxins, and cytotoxin-related genes. Studies have shown that the prevalence of *Helicobacter pylori* infection in the general population is more than 50% [1]. It is recognized as the culprit of chronic gastritis, gastric ulcers, and gastric cancer. In addition to causing the gastrointestinal disease, it is also thought to have extragastrointestinal effects, including idiopathic thrombocytopenia purpura, chronic idiopathic urticaria, iron deficiency anemia, arteriosclerosis, and ischemic heart disease [2–4].

In recent years, several studies demonstrated that *Helicobacter pylori* infection is involved in metabolic syndrome which is composed of the following major components: abdominal obesity, insulin resistance (IR), elevated BP, and dyslipidemia [5].

The colorectal adenomatous polyp is a common disease of the digestive system and is closely related to the occurrence of colorectal cancer. Studies have confirmed that metabolic syndrome is a risk factor for colorectal cancer [6]. To further explore the relationship between *Helicobacter pylori* infection and colorectal adenomatous polyps, we observed the relationship between *Helicobacter pylori* infection and colorectal adenomatous polyps by means of electronic colonoscopy.

## 2. Material and Method

### 2.1. Study Population and Data Collection

This study was conducted at Sunshine Union Hospital. We followed the methods of Yang et al. [7]. All the qualified subjects who took part in the study were hospitalized from January 2019 to July 2019. Inclusion criteria were complete, available medical records and having gastroscopy in our hospital; having not used proton pump inhibitors (PPIs), histamine type 2 receptor antagonists (H2A), antibiotics, bismuth, or sucralfate for up to one month prior to the endoscopy; normal blood pressure (diastolic blood pressure (DBP) of less than 90 mmHg and systolic blood pressure (SBP) of less than 150 mmHg); or well-controlled hypertension (DBP of less than 90 mmHg and SBP of less than 150 mmHg). Exclusion criteria were the presence of hematological diseases; past history of cancer; major gastrointestinal surgery, including partial or total gastrectomy or colectomy; pulmonary disease; diabetes mellitus; smoking; alcohol consumption; kidney diseases; pregnancy and lactation; and having been using medicine which is known to be able to effect blood coagulation, such as aspirin and clopidogrel. Written informed consent was obtained from each subject and was recorded by the physician who explained the study procedures. The study was reviewed and approved by the Ethics and Research Committee of Sunshine Union Hospital (Shandong, China), and the reported investigations were carried out in accordance with the principles of the Declaration of Helsinki as revised in 2000.

#### 2.1.1. Diagnosis of Colorectal Polyps

The operations of colonoscopy were performed by physicians who had been trained for 6 months or longer at our hospital and had carried out 500 or more colonoscopies. Insertion up to the cecum was successful in 96.8% of examinations. Anal and rectal finger examination was the routine prior to insertion of the endoscope. After arriving at the end of the ileum, slowly exit the colonoscopy and carefully observe the colorectal wall mucosa, and retreat time is not less than 6 minutes. In preparation for colonoscopy, subjects took a laxative (polyethylene glycol electrolyte powder, Beaufour Ipsen Industrie, Dreux, France) on the night before the examination and on the day of colonoscopy at 08:30 h (colonic lavage using approximately 2000 mL of solution was carried out). Small-caliber electronic colonoscopies (CF-200I, 240I, 240AI, and Q260AI; Olympus, Tokyo, Japan) were used for colonoscopy. All visualized lesions were biopsied and histologically assessed by experienced pathologists.

### 2.2. *Helicobacter pylori* Infection Test

The diagnosis of *Helicobacter pylori* infection was based on the results of the fasting ^13^C urea breath test (^13^C-UBT). Breath tests were conducted by dedicated nurses. Subjects fasted overnight and maintained normal exhalation. A straw was inserted into the bottom of a sample tube. The straw was slowly inhaled into the sample tube for 4 to 5 seconds, and the straw was pulled out. Immediately tighten the test tube cover. This method collects 0 minute of exhalation. The patient took a bottle of urea ^13^C particles with 80 to 100 mL cold drinking water and sat still. According to the above method of collecting exhalation, the patient collected 30 minutes of exhalation after taking urea ^13^C and twisted the test tube cover. The 0-minute and 30-minute exhalation sample tubes collected will be tested on the corresponding instrument for ^13^C O_2_ (the reagent kit will be provided by Beijing Buzhan Pharmaceutical Co., Ltd., and the instrument will be provided by Beijing Huayuan Anbang Technology Co., Ltd—type ^13^C infrared spectrometer). The ^13^C urea exhalation test was positive for *Helicobacter pylori* infection as the delta over baseline is greater than or equal to 4.0, and the ^13^C urea exhalation test was negative for *Helicobacter pylori* infection as the delta over baseline is less than 4.0.

### 2.3. Biochemical Analyses

Blood samples were taken into anticoagulated tubes from participants after an overnight fast of more than 12 h. Plasma was separated by centrifugation at 3000 × *g* for 10 minutes at room temperature. The levels of creatinine, BUN, total cholesterol, triglyceride, and glucose were measured using a multichannel analyzer (Roche Hitachi 737; Boehringer Mannheim Diagnostics, USA).

### 2.4. Statistical Analysis

Data were expressed as mean ± SD or counts. Statistical analysis was performed using SPSS version 16.0 (SPSS Inc., Chicago, IL), and the level of statistical significance was defined as *P* < 0.05. The independent samples *t*-test was used for the comparisons of continuous data, while the chi-square test was used for the comparisons of categorical variables. Binary logistic regression analysis was used to determine the factors that were associated with colorectal adenomatous polyps.

## 3. Results

### 3.1. Comparison of Variables in the Healthy Control and the *Helicobacter pylori* Infection Group

Comparisons of the variables between the healthy control and the *Helicobacter pylori* infection group are shown in Table 1. Patients with *Helicobacter pylori* infection had higher incidence of colorectal adenomatous polyps and multiple colorectal adenomatous polyps, higher levels of total cholesterol and fasting glucose, and more males (*P* < 0.05, *P* < 0.01).

### 3.2. *Helicobacter pylori* Infection and Risk Factors for Colorectal Adenomatous Polyps

Binary logistic regression analysis was used to evaluate the risk factors for colorectal adenomatous polyps. Colorectal adenomatous polyps were taken as the dependent variable, and age, gender, *Helicobacter pylori* infection, total cholesterol, triglyceride, fasting glucose, and hypertension were taken as independent variables. It was found that *Helicobacter pylori* infection (*P* < 0.05, OR 2.383) was significantly associated with the risk of colorectal adenomatous polyps (Table 2).

## 4. Discussion

Colorectal adenomatous polyps can be considered precancerous lesions, for hyperproliferative epithelial cells can transform from the adenomatous stage to the colorectal cancer stage [8, 9]. Early diagnosis of adenomatous polyps depends on colonoscopy. With the popularity of colonoscopy, colorectal adenomatous polyps were discovered early and removed in time. The pathogenesis of colorectal adenomatous polyps remains unclear. From the results of this study, it is found that *Helicobacter pylori* infection is related to colorectal adenomatous polyps, which is in agreement with the previous studies [10, 11].

The exact mechanism of *Helicobacter pylori* infection and colorectal adenomatous polyps is not yet clear. The following mechanisms may help explain our findings. First, *Helicobacter pylori* infection could upregulate the expression of matrix metalloproteinases [12], which may take part in not only colorectal carcinogenesis from adenomatous polyps but also colorectal tumor invasion and initiation of a metastatic cascade [13]. Second, *Helicobacter pylori* infection can lead to abnormal secretion of ghrelin secreted by the gastric mucosa [14], and ghrelin can prevent the occurrence of colorectal adenomatous polyps and colorectal cancer [15]. Third, *Helicobacter pylori* effects the inflammatory state of the intestine through the release of cytotoxins, lipopolysaccharide (LPS), and other toxic substances [16], which are involved in the formation of colorectal adenomatous polyps [17].

In short, from the results of this study, we can see that *Helicobacter pylori* infection is associated with the occurrence of colorectal adenomatous polyps, so the treatment of *Helicobacter pylori* infection may help prevent colorectal adenomatous polyps.

The shortcomings of this study include the following: first, the number of sample cases observed is small; secondly, the subjects selected for observation are those who go to the hospital for medical treatment or health examination. Furthermore, there was no in-depth study on the internal mechanism of increased incidence of *Helicobacter pylori* infection and colorectal adenomatous polyps.

## Figures and Tables

**Table 1:** Comparison of the variables of patients in the healthy control and *Helicobacter pylori* infection group.

Variables	Healthy control (*n* = 98)	*Helicobacter pylori* infection (*n* = 68)	*P* value
Mal, *n* (%)	42 (62.86)	45 (66.18)	0.003
Age	53.551 ± 9.276	54.279 ± 9.098	0.617
Colorectal adenomatous polyps, *n* (%)	30 (30.61)	36 (52.94)	0.004
Multiple colorectal adenomatous polyps, *n* (%)	13 (13.26)	27 (39.70)	0.001
Single colorectal adenomatous polyp, *n* (%)	8 (8.16)	7 (10.29)	0.639
Total cholesterol (mmol/L)	4.564 ± 1.586	5.152 ± 1.054	0.008
Triglycerides (mmol/L)	1.477 ± 1.131	1.833 ± 1.625	0.098
Hypertension, *n* (%)	12 (12.24)	13 (19.12)	0.225
Fasting glucose (mmol/L)	4.978 ± 1.796	5.593 ± 1.417	0.019

**Table 2:** The results of binary logistic regression analysis of colorectal adenomatous polyps.

		Beta	SE	*P* value	OR	95% CI	
						Lower	Upper
Mal, *n*	Female = 0, male = 1	0.278	0.342	0.417	1.321	0.675	2.583
Age, years	<60 = 0, ≥60 = 1	0.084	0.382	0.826	1.088	0.514	2.301
*Helicobacter pylori* infection, *n*	Absent = 0, present = 1	0.868	0.344	0.012	2.383	1.214	4.676
Total cholesterol (mmol/L)	<5.69 = 0, ≥5.69 = 1	0.140	0.359	0.696	1.151	0.569	2.327
Triglycerides (mmol/L)	<1.7 = 0, ≥1.7 = 1	-0.209	0.373	0.575	0.811	0.391	1.685
Hypertension, *n*	Absent = 0, present = 1	0.277	0.481	0.564	1.319	0.514	3.385
Fasting glucose (mmol/L)	<6.1 = 0, ≥6.1 = 1	-0.092	0.454	0.839	0.912	0.375	2.220

## Data Availability

The data used to support the findings of this study are available from the corresponding author upon request.
